# Chest Pain from Pneumopericardium with Gastropericardial Fistula

**DOI:** 10.1155/2021/5143608

**Published:** 2021-07-14

**Authors:** Abdullah Rathur, Hussein Al-Mohamad, Jeffrey Steinhoff, Ronald Walsh

**Affiliations:** Department of Cardiology, HCA Healthcare/USF Morsani College of Medicine GME Programs/Largo Medical Center, Largo, FL, USA

## Abstract

**Introduction:**

Gastropericardial fistula, a connection between the upper gastrointestinal tract and pericardium, is a rare clinical finding most commonly associated with postsurgical complications, as well as direct tissue invasion from gastric cancer. *Case Report*. We report a case of a 58-year-old Caucasian woman with metastatic colon cancer treated with FOLFOX, a combination chemotherapy regimen, and bevacizumab who presented with chest pain. She was ruled out for acute coronary syndrome, aortic dissection, or pulmonary embolism. A computed tomography (CT) scan of her chest showed pneumopericardium. A barium swallow ruled out esophageal ulceration, and esophagogastroduodenoscopy (EGD) showed a large penetrating gastric ulcer with no evidence of gastric dysplasia or malignancy or evidence of Helicobacter pylori (H. pylori). The patient underwent median sternotomy with gastric ulcer resection and repair, as well as pericardial washout and pericardial chest tube placement. After an uncomplicated course, she was safely discharged home.

**Conclusion:**

Given that gastrointestinal ulceration and perforation are known phenomena in patients taking vascular endothelial growth factor (VEGF) inhibitors, surveillance endoscopy may be beneficial to discover them before they result in potentially fatal complications such as gastropericardial fistulas.

## 1. Introduction

Pneumopericardium is the presentation of air within the pericardial cavity and can be caused by trauma, thoracic surgery, thoracentesis, infectious pericarditis, foreign body aspiration, cocaine inhalation, and fistula formation between the pericardium and other structures [1]. Gastropericardial fistula, a rare condition caused by pathological communication between the upper gastrointestinal tract and the pericardium, is one of the causes of pneumopericardium. The gastropericardial fistula is associated with significant morbidity and mortality, reaching as high as 69% in one study [2]. The presentation of gastropericardial fistulas varies, with acute dyspnea, chest pain, hemodynamic instability from cardiac tamponade, or even cardiac arrest from tension pneumopericardium [3]. Conditions most commonly associated with gastropericardial fistulas include prior gastroesophageal surgery such as Nissen fundoplication or hernia repair, gastroesophageal cancer [2], and even ulcers associated with gastroesophageal reflux disease, NSAID use, and Zollinger-Ellison syndrome [4–6]. This case highlights a very rare diagnosis of gastropericardial fistula in a patient on VEGF-inhibitor chemotherapy for metastatic colon cancer, in the absence of gastric cancer, prior gastroesophageal surgery, or significant risk factors for peptic ulcer disease.

## 2. Case Presentation

A 58-year-old Caucasian woman with a past medical history of stage IV colon cancer being treated actively with FOLFOX and bevacizumab presented to the emergency department (ED) with complaint of left-sided chest pain and left shoulder pain which started over a year ago but acutely increased in intensity and frequency over the past three weeks. The pain was inconsistently worse with position, associated with dyspnea, left-sided neck pain, and belching, without fever, chills, cough, abdominal pain, nausea, vomiting, diarrhea, dysphagia, melena, or hematochezia. Her last chemotherapy treatment was five days prior. She was initially diagnosed with the cancer 2.5 years ago with metastasis to the liver and was treated initially with diverting colostomy and chemotherapy with bevacizumab, later modified to include FOLFOX. The patient denied nonsteroidal anti-inflammatory drug (NSAID) use or alcohol or illicit drug abuse.

**Figure 1:**
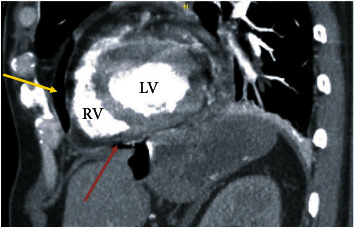
Sagittal view of chest CT showing pneumopericardium (yellow arrow) and gastropericardial fistula (red arrow).

In the ED, initial vital signs showed blood pressure 174/83 mmHg, heart rate 101/minute, respiratory rate 24/minute, SpO_2_ 97% on room air, temperature 96.9°F. Physical examination was unremarkable except for decreased breath sounds and regular tachycardia. Labs showed hemoglobin (16 g/dL), alkaline phosphatase (299 U/L), and negative urine drug screen. Serial troponins six hours apart were negative, and initial ECG showed sinus rhythm without ischemic changes. Thoracic CT angiogram ruled out pulmonary embolism and showed pneumopericardium, gastropericardial fistula, and three liver lesions suspicious for metastatic disease, as shown in Figure 1.

A transthoracic echocardiogram showed a left ventricular (LV) ejection fraction of 55-60%, no valvular disease, and a normal pulmonary pressure and suggested the presence of pericardial air due to significant acoustic attenuation in otherwise previously normal windows. A barium swallow was performed with no visible esophageal stricture or ulceration, but the field of view did not include the stomach.

An EGD was done and showed a normal esophagus and 3 cm penetrating gastric cardia ulcer with surrounding edema and nonbleeding vessel in the center; biopsy was negative for dysplasia, metaplasia, malignancy, or H. pylori.

Surgical intervention was performed by cardiothoracic and general surgery teams, including median sternotomy, laparotomy, intraoperative transesophageal echocardiogram (TEE) to delineate the area of ulceration, posterior pericardial debridement of tissue adhesions, gastric ulcer resection and primary repair with omental patch, gastrojejunostomy feeding tube insertion, diaphragmatic patch with mesh, pericardial washout, and pericardial/pleural chest tube placement. She was transferred to the intensive care unit (ICU) and placed on total parenteral nutrition. After a few days, she was discharged home without any postoperative complications.

## 3. Discussion

Gastropericardial fistula is a rare condition typically associated with prior gastroesophageal surgery or gastroesophageal cancer [2]. Presentation is highly variable, ranging from benign left shoulder pain to cardiac arrest from tension pneumopericardium [3].

Our patient presented with chest pain and long-standing left shoulder pain, initially attributed to rotator cuff pathology. However, long-standing shoulder pain is an important symptom associated with nontraumatic gastric perforation into the pericardium [7], and chest or left shoulder pain is the presenting symptom in 66% of cases of gastropericardial fistula [2]. Overall, the prognosis is typically poor with this condition due to late recognition in an acute chest pain setting. Gastropericardial fistula is frequently associated with infection and sepsis, requiring surgical intervention for repair [8, 9]. Pneumopericardium is often first diagnosed by chest X-ray, but the finding is nonspecific in identifying the etiology. Contrast CT imaging is useful for identifying a fistulous tract between the GI tract and the pericardium [3]. Endoscopy allows diagnosis and localization of a fistula by direct visualization and is beneficial in those unidentified by contrast imaging, and it also allows for biopsy of the ulcer to look for malignancy. Per literature review by Davidson et al., fluoroscopic contrast studies to detect gastropericardial fistula should be reserved for cases with negative initial CT or in equivocal cases. Transthoracic echocardiography is not usually effective for diagnosing pneumopericardium due to air attenuation artifact [10].

Gastrointestinal perforation is a known side effect of bevacizumab, a humanized monoclonal antibody against the vascular endothelial growth factor (VEGF). Vasoactive agents such as prostaglandins and nitrous oxide are activated by VEGF, and inhibition of growth factors can lead to weakened mucosal defense [11]. Animal models showed the regression of capillaries of intestinal villi which could contribute to perforation if exacerbated by other pathologic processes [12]. In a study by Hendrick et al., 1.7% of patients being concomitantly treated with bevacizumab and first-line chemotherapy had gastrointestinal perforation, with 68% occurring in the first 60 days. Our literature review did not show any prior reported cases of gastropericardial fistula in the setting of VEGF-inhibitor usage without any other risk factors.

Gastropericardial fistula in the absence of gastric malignancy, prior gastrointestinal surgery, or combination chemotherapy and radiation therapy is a very rare clinical syndrome. In one previously reported case, the patient had no prior gastric malignancy or surgery, and the only risk factor for gastric ulceration was alcohol abuse [13], which has been shown to be a risk factor for ulcer perforation [13]. Despite having metastatic colon cancer, our patient's gastric biopsy was negative for malignancy or H. pylori, and she denied a history of significant NSAID or alcohol use, making peptic ulcer disease a less likely cause. Gastropleural, gastropericardial, and gastropleuropericardial fistula formations have been reported with combination radiation therapy and chemotherapeutic agents such as fluorouracil, oxaliplatin, and sunitinib [14]. Radiation treatment results in vessel destruction and tumor necrosis, causing endothelial cytotoxicity and potential adhesion between the gastric fundus and diaphragm, leading to possible gastropericardial fistula formation [15–17]. As per standard of care, our patient did not need to receive radiation therapy. Pericardial metastasis can cause pericardial effusion, clinical pericarditis, and sometimes tamponade, but this was not the case in our patient as per biopsy. Again, based on literature review, our case appears to be the first reported case of gastropericardial fistula as a result of VEGF-inhibitor use in a patient without prior gastroesophageal surgery, invading gastroesophageal or pericardial cancer, or any significant risk factors for peptic ulcer disease.

## 4. Conclusion

Clinical presentation of gastropericardial fistulas can vary from mild chest pain and shortness of breath to cardiac arrest from tension pneumopericardium, and there is usually little to no clinical suspicion for such an uncommon diagnosis. Given that this clinical diagnosis can be difficult, a more useful approach may be prevention before a gastropericardial fistula can form. Although there are currently no such guidelines, it may be prudent to consider surveillance endoscopy in patients taking combination VEGF inhibitors and other chemotherapeutic agents to assess for gastrointestinal ulceration in order to intervene before complications like gastropericardial fistula can occur.

## Supplementary Materials

Supplementary Materials Included in the supplementary files are additional CT images of the pneumopericardium in the transverse section (Figure S1) and gastropericardial fistula in the coronal section (Figure S2).
