# Supplementary figures and images for: Chest Pain from Pneumopericardium with Gastropericardial Fistula
Source: Case Rep Cardiol. 2021 Jul 14;2021:5143608. doi: 10.1155/2021/5143608 (PMC8294994; doi:10.1155/2021/5143608)

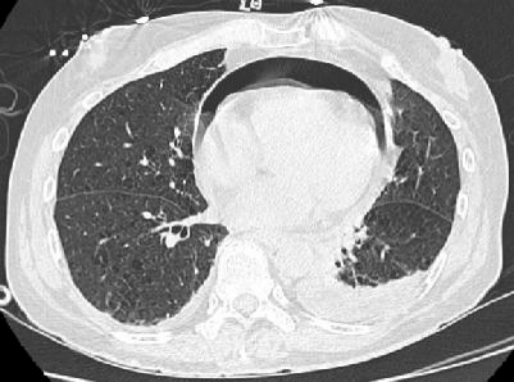

Supplement: Supplementary Materials — Included in the supplementary files are additional CT images of the pneumopericardium in the transverse section (Figure S1) and gastropericardial fistula in the coronal section (Figure S2). [file 5143608.f1.zip › Figure S1.pdf]

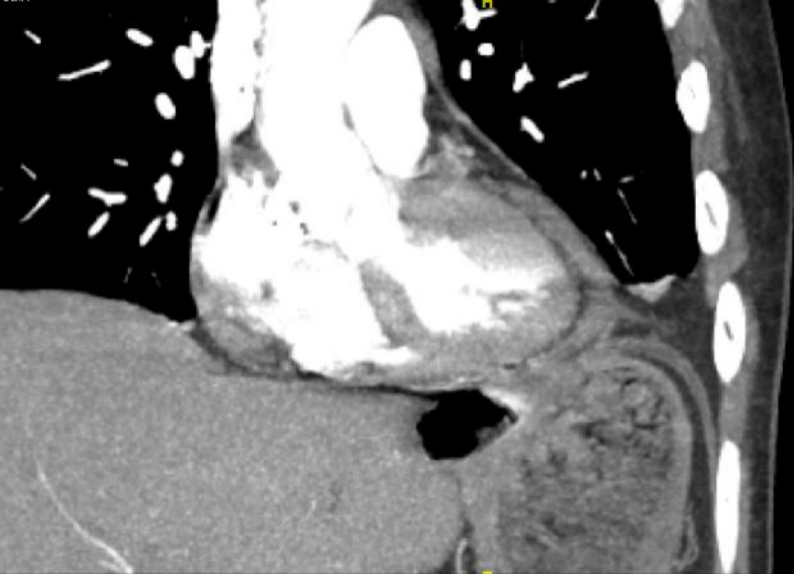

Supplement: Supplementary Materials — Included in the supplementary files are additional CT images of the pneumopericardium in the transverse section (Figure S1) and gastropericardial fistula in the coronal section (Figure S2). [file 5143608.f1.zip › Figure S2.pdf]
